# Dissolved oxygen levels differentially regulate ammonia metabolism plasticity in mandarin fish (*Siniperca chuatsi*)

**DOI:** 10.3389/fphys.2026.1912438

**Published:** 2026-08-04

**Authors:** Hongyan Li, Jiaqi Xu, Guangjun Wang, Zhifei Li, Kai Zhang, Jingjing Tian, Yun Xia, Wenping Xie, Quanfa Zhong, Jun Xie, Wangbao Gong

**Affiliations:** 1 Key Laboratory of Tropical & Subtropical Fishery Resource Application & Cultivation, Pearl River Fisheries Research Institute, Chinese Academy of Fishery Sciences, Guangzhou, China; 2 Graduate School of Chinese Academy of Agricultural Sciences, Beijing, China

**Keywords:** ammonia excretion, dissolved oxygen, mandarin fish, metabolic plasticity, nitrogen management

## Abstract

Dissolved oxygen (DO) is a key environmental factor affecting fish metabolism, yet its regulatory role in ammonia production, conversion, and excretion remains poorly understood. This study investigated how DO levels modulate ammonia metabolism plasticity in mandarin fish (*Siniperca chuatsi*) as a potential biological strategy for mitigating aquaculture-derived ammonia pollution. Fish were exposed to normoxia (6.05 ± 0.17 mg/L), hypoxia (2.67 ± 0.25 mg/L), or hyperoxia (9.73 ± 0.62 mg/L) for 48 h. Results showed that hypoxia caused oxidative stress, as evidenced by elevated MDA levels, suppressed SOD and CAT activities, and obvious tissue damage to the livers and gills of *S. chuatsi*. In response to this stress, both hypoxia and hyperoxia enhanced the antioxidant capacity of *S. chuatsi* via the Nrf2/Keap1 pathway. Compared to the normoxia and hyperoxia groups, the ammonia excretion rate was significantly lower in the hypoxia group. Distinct adaptive strategies in ammonia homeostasis were observed under different DO conditions. Under hypoxic condition, *S. chuatsi* downregulated hepatic glutamate dehydrogenase (*gdh*) to reduce ammonia production, upregulated carbamyl phosphate synthase 1 (*cps1*) to promote ammonia conversion to urea, and upregulated RH glycoproteins and Na^+^/H^+^ exchanger 3 (*nhe3*) as a compensatory response, ultimately leading to a 24.06% reduction in water NH_4_^+^-N accumulation at 48 h. In contrast, hyperoxia reduced ammonia production and increased urea conversion but did not enhance excretion. Instead, it improved ammonia tolerance in *S. chuatsi*. In summary, hypoxia reduced net ammonia excretion via integrated adjustments in production, conversion, and excretion, whereas hyperoxia enhanced ammonia tolerance without altering excretion. This DO-mediated metabolic plasticity provides a biological strategy for mitigating aquaculture-derived ammonia pollution by utilizing existing aeration to harness intrinsic fish physiology, supporting the development of sustainable aquaculture.

## 1 Introduction

Aquaculture is a vital contributor to human protein supply and global food security, accounting for approximately 60% of the world’s total aquatic product output (FAO, 2024). China is the world’s largest aquaculture producer, contributing over 60% of global aquaculture production (FAO, 2024). However, the rapid expansion of intensive aquaculture in China has also arouse significant environmental impacts, among which ammonia pollution poses a major threat to aquatic ecosystem integrity and constrains the sustainable development of the aquaculture industry (Chen et al., 2023; Timmons and Ebeling, 2013). Aquaculture-derived ammonia (including the ionized NH4^+^ form and unionized NH3) primarily originates from fish metabolic excreta and the decomposed residual feed (Liu et al., 2019). Excessive accumulation of ammonia triggers eutrophication and promotes harmful algal blooms, reduces dissolved oxygen (DO) via nitrification, which ultimately poisoning the aquatic organisms (Edwards et al., 2024). Moreover, ammonia (especially NH_3_) toxicity suppresses the growth, immunity, reproduction, and cause tissue damage in fish (Barbieri et al., 2019; Liu et al., 2021), which constrains the healthy development of aquaculture industry.

To address aquaculture-derived ammonia pollution, several strategies have been employed to remove ammonia in the environment through physical and chemical methods (Hargreaves, 2013; Yang et al., 2022). However, these conventional approaches are undertaken to mitigate ammonia pollution after it has been generated (Cao et al., 2025). They are often limited by high operational costs, low sustainability, and the risk of secondary pollution. Moreover, the efficiency of physicochemical approaches varies considerably across different aquaculture systems, which highlight the need for alternative strategies that prevent ammonia generation at its source rather than removing it after production. Consequently, biological strategies, which utilize the intrinsic metabolic capacity of organisms to manage pollutant levels, could be of great significance. Fish exposed to ammonia stress exhibit species-specific detoxification strategies: while some species prioritize oxidative stress mitigation via Nrf2/glutathione redox regulation, others enhance urea cycle enzyme activities to convert ammonia to urea (Yu and Liu, 2026). As the core biological component of aquaculture systems, teleost fish play a decisive role in ammonia cycling through their ammonia metabolism and excretion processes (Ip and Chew, 2010a). Modulating fish metabolic processes to reduce ammonia release has become a promising direction for mitigating aquaculture waste.

Ammonia metabolism in fish is a comprehensive process involves three key interlinked processes: production, conversion, and excretion (Ip and Chew, 2010a; Parvathy et al., 2023). Numerous physiological activities in fish could produce ammonia, with the catabolism of amino acids being the predominant pathway for source of ammonia within fish bodies (Mommsen and Walsh, 1992). The liver is a central organ for ammonia generation (Ip and Chew, 2010a), where glutamate dehydrogenase (GDH) plays a critical role in the deamination of glutamate (Ballantyne, 2001). In addition, adenosine monophosphate deaminase (AMPD) in muscle tissue serves as a major source of ammonia under conditions of high energy demand, such as hypoxia or exercise (Ip and Chew, 2010a). To mitigate the inherent toxicity of accumulated ammonia, fish have evolved sophisticated detoxification mechanisms. Accumulated ammonia could be converted into less toxic compounds such as urea via the ornithine-urea cycle (OUC) or glutamine via glutamine synthesis (Zhang et al., 2022). Carbamyl phosphate synthase I (CPS I) is the rate-limiting enzyme in the OUC pathway in mammals (Neill et al., 2009). Additionally, the gills serve as the major excretory organ for nitrogenous wastes in fish, responsible for up to 90% of total ammonia excretion (Ip and Chew, 2010b). The excretion of ammonia in the gills is not solely reliant on passive diffusion of NH_3_ but is actively facilitated by specialized transporters, including Rhesus (RH) glycoproteins (e.g., Rhag, Rhbg, Rhcg) and the Na^+^/H^+^ exchanger 3 (NHE3), which collectively mediate the efficient ammonia transfer across the branchial epithelium (Hoseini et al., 2024; Zhao et al., 2024). Comparative studies in aquatic invertebrates have also provided valuable insights into the conserved mechanisms of ammonia excretion (Weihrauch and Allen, 2018).

Additionally, dissolved oxygen (DO) is a critical abiotic factor regulating fish physiology and metabolism. In intensive aquaculture systems, DO fluctuations—ranging from hypoxia to hyperoxia—are common due to uneven aeration, high stocking densities, and diurnal algal respiration. Although existing evidence indicates that DO levels can influence both ammonia metabolism and its toxicity in fish, the regulatory mechanisms remain poorly understood (Parvathy et al., 2023). In the present study, DO-mediated metabolic plasticity was defined as the ability of fish to dynamically adjust ammonia production, conversion, and excretion pathways in response to changing DO levels. Furthermore, the potential of leveraging DO-mediated plasticity in ammonia metabolism as a sustainable, biological strategy for mitigating aquaculture-derived ammonia pollution has not been systematically explored.

Mandarin fish (*Siniperca chuatsi*) is one of the most economically important freshwater fish widely cultivated in China, with annual production exceeding 539,000 tons, and is increasingly cultivated in Southeast Asian countries due to its high market value (Ministry of Agriculture and Rural Affairs of the People's Republic of China, Bureau of Fisheries, 2025; Li et al., 2023). Notably, mandarin fish exhibits highly sensitivity to fluctuations in dissolved oxygen (Li et al., 2025), making it an ideal model for ammonia metabolism analysis in response to DO manipulation. Existing studies on *S. chuatsi* have focused on oxidative stress and transcriptome adaptations under hypoxia conditions (Gong et al., 2026), but a comprehensive understanding of how ammonia metabolism (production, conversion, and excretion) responds to varying DO levels remain lacking. To address these gaps, this study exposed *S. chuatsi* to normoxia, hypoxia, and hyperoxia conditions, systematically investigating the changes in antioxidant capacity and ammonia metabolism-related indices at the physiological and molecular levels. We hypothesized that DO levels modulate ammonia metabolism through integrated adjustments in production, conversion, and excretion, with the direction and magnitude of these adjustments differing between hypoxia and hyperoxia. To our knowledge, this is the first study to systematically examine the three interconnected processes of ammonia metabolism—production, conversion, and excretion—in response to different DO levels in fish, and to evaluate DO-mediated metabolic plasticity as a biological strategy for mitigating ammonia pollution.

## 2 Materials and methods

### 2.1 Experimental fish acclimation and DO manipulation

Healthy mandarin fish (*Siniperca chuatsi*) with uniform body size (57.51 ± 2.91 g) were obtained from a local rearing farm in Foshan, Guangdong Province, China. Prior to the experiment, the fish were acclimated for two weeks in a recirculating aquaculture system. During the acclimation, all fish were fed live prey fish twice daily (at 08:00 and 18:00) to satiation. Fish were fasted for 24 h prior to the experimental exposure.

Three dissolved oxygen (DO) levels were established: normoxia (6 mg/L), hypoxia (3 mg/L), and hyperoxia (9 mg/L). The exposure experiment was conducted in a non-recirculating system, with three replicate tanks per group. DO levels were regulated by aerating air, pure oxygen, or nitrogen into the water in 240 L cylindrical tanks contained 15 fish, corresponding to a stocking density of approximately 3.6 kg/m³. Fish were fasted during the 48-h exposure to avoid dietary interference with ammonia metabolism measurements. DO levels were monitored every 2 h using a YSI Professional Plus portable water quality analyzer (YSI Professional Plus, USA) to maintain stability throughout the 48-h exposure period. The exposure commenced (recorded as 0 h) once the target DO levels had been reached and stabilized in all groups. Water temperature was maintained at 28 ± 1.5 °C, and pH was measured every 6 h at a value of 7.86 ± 0.21. Throughout this manuscript, hypoxia, normoxia, and hyperoxia refer to the operational DO conditions used in this study, with measured concentrations of 2.67 ± 0.25, 6.05 ± 0.17, and 9.73 ± 0.62 mg/L, respectively, rather than to universal physiological thresholds.

### 2.2 Sampling

Water samples were collected from each tank at 2-hour intervals, after which they were stored at -20 °C for subsequent water quality analysis. For tissue sampling, three fish per tank were randomly selected at 0 h, 24 h, and 48 h, respectively. Fish were euthanized by a sharp blow to the head under deep MS-222 anesthesia (50 mg/L, Sigma-Aldrich, USA) prior to dissection. Plasma was obtained by drawing blood from the caudal vein using a sterile syringe. The plasma was then centrifuged, and the supernatant was aliquoted and stored at –80 °C. Liver, skeletal muscle, and gill tissues were rapidly dissected on an ice-chilled plate. All tissue samples were immediately snap-frozen in liquid nitrogen and stored at –80 °C until subsequent assays.

### 2.3 Determination of water quality indices and ammonia excretion rate

Prior to analysis, water samples were filtered through a 0.45 μm pore-size polyethersulfone syringe filter. Key nitrogen and phosphorus parameters were determined using standard spectrophotometric methods, as summarized in **Table 1**. The ammonia excretion rate was calculated using the following formula:

**Table 1:** Methods for determination of water quality parameters.

Parameter	Method
Total nitrogen (TN)	Alkaline potassium persulfate digestion-UV spectrophotometry
Nitrate nitrogen (NO_3_^-^-N)	Thymol spectrophotometry
Nitrite nitrogen (NO_2_^-^-N)	N-(1-naphthyl)-ethylenediamine spectrophotometry
Ammonium nitrogen (NH_4_^+^-N)	Salicylic acid spectrophotometry
Phosphate (PO_4_³^-^-P)	Molybdenum blue method
Total phosphorus (TP)	Ammonium molybdate spectrophotometry


$$A_{ER}=\frac{{\left[T_{amm}\right]}_{f}-{\left[T_{amm}\right]}_{i}\times V}{\Delta t\times m}$$

where A_ER_ denotes the ammonia nitrogen excretion rate (μmol(N)/(kg·h)); [Tamm]_i_ and [Tamm]_f_ represent the initial and final total ammonia nitrogen concentrations (μmol(N)/L), respectively; V denotes the water volume (L); Δt denotes the time difference between the start and end of excretion; m denotes body weight (kg). The phosphorus excretion rate was calculated using the following formula:


$$P_{ER}=\frac{{\left[T_{P}\right]}_{f}-{\left[T_{P}\right]}_{i}\times V}{\Delta t\times m}$$

where P_ER_ denotes the phosphorus nitrogen excretion rate (μmol(P)/(kg·h)); [T_P_]_i_ and [T_P_]_f_ represent the initial and final phosphorus concentrations (μmol(P)/L), respectively; V denotes the water volume (L); Δt denotes the time difference between the start and end of excretion; m denotes body weight (kg).

### 2.4 Metabolites and enzyme activities in plasma and liver

Liver tissues were weighed and homogenized on ice in a ratio of nine volumes (w/v) of normal saline to one volume of tissue to obtain 10% (w/v) homogenates. The homogenates were then centrifuged at 3000 × g for 10 min at 4 °C, and the resulting supernatants was collected. Plasma urea (PU) concentration was determined using a commercial assay kit (C013-3-1, Nanjing Jiancheng Bioengineering Institute, China). Ammonia levels in plasma and liver homogenates were quantified using a commercial kit (A086-2-1, Nanjing Jiancheng Bioengineering Institute, China) based on the salicylic acid spectrophotometric method for total ammonia nitrogen determination. Hepatic antioxidant status was assessed by measuring total antioxidant capacity (T-AOC), superoxide dismutase (SOD) activity, catalase (CAT) activity, and malondialdehyde (MDA) content using corresponding commercial kits (Nanjing Jiancheng Bioengineering Institute, China). All procedures were performed in accordance with the manufacturers’ instructions.

### 2.5 Histopathological analysis

Fresh liver and gill tissue samples were fixed in a 4% paraformaldehyde solution for over 24 hours. The fixed tissues were then dehydrated through a graded ethanol series, cleared in xylene, and embedded in paraffin wax. Sections of 4 µm thickness were obtained using a rotary microtome. After deparaffinization and rehydration, the sections were stained with hematoxylin and eosin (H&E) according to standard protocols. Finally, the stained sections were mounted with neutral gum and examined under a light microscope for histological observation.

### 2.6 Total RNA extraction and quantitative real-time PCR (qPCR)

Total RNA was extracted from liver, gill, and muscle tissues using the MagaBio plus Total RNA Purification Kit II (Hangzhou Bioer Technology Co., Ltd., China) following the manufacturer’s protocol. The concentration and purity of total RNA were detected using a Implen NanoPhotometer (Implen Inc., Germany), with A260/A280 ratios between 1.8 and 2.0. Then, cDNA was synthesized by reverse transcription using the PrimeScript™ RT reagent Kit (TaKaRa, Japan). Primers for target genes and the internal reference gene (β-actin) were present in **Table 2**. The specificity of primers was verified by agarose gel electrophoresis and melting curve analysis. qPCR was performed on a LightCycler^®^ 96 Instrument (Roche, Switzerland). TB Green^®^ Fast qPCR Mix (TaKaRa, Japan) was used in the 20 μL-reaction volume, including 10 μL TB Green, 0.8 μL forward primer (10 μmol/L), 0.8 μL reverse primer (10 μmol/L), 2 μL cDNA template (100 ng/μL), and 6.4 μL ddH_2_O. The amplification program was: 95°C for 5 min (pre-denaturation, 1 cycle); 95°C for 15 s (denaturation), 60°C for 1 min (annealing and extension), 45 cycles; 50°C for 30 s (melting curve, 1 cycle). The relative expression levels of genes were calculated using the 2^(-ΔΔCt) method.

**Table 2:** Primers used in the present study.

Gene		Primer sequence (5’-3’)	Accession No.
*gdh*	F:	GACGACGACCCCAACTTCT	XM_044177925.1
	R:	GACCCGCTTCCTCTTCTGC	
*cps1*	F:	ACACTTGGCGAACTACAGCA	XM_044215566.1
	R:	AGAATCTGACCAACCTGCCG	
*nrf2*	F:	ACGAAAGCGAAAGCTCCTCA	XM_044216334.1
	R:	GCTCTCTTCCAGAATGGCGT	
*keap1*	F:	GTGGCAACCCAGGAGGAG	XM_044189604.1
	R:	GGGAATGGCAACGGACA	
*gr*	F:	CAGGCATCCTTTCCACCC	XM_044204920.1
	R:	TCCAGTCCTCTGTCCGTTTTA	
*sod*	F:	CACGCTCCCTGACCTGACA	XM_044168058.1
	R:	GGAGGGCAACCTGTGCTG	
*cat*	F:	GCGTTTGGCTACTTTGAGGT	XM_044194118.1
	R:	CACAGTGGAGAAGCGGACA	
*gpx*	F:	GCCCATCCCCTGTTTGTG	XM_044172415.1
	R:	AACTTCCTGCTGTAACGCTTG	
*ampd*	F:	CATTTCCTTCCCGTGTT	XM_044210936.1
	R:	TCTGTCTGCGGAGTTGGT	
*gls1b*	F:	TTGGAAAAAGGTGTCCCCCA	XM_044217470.1
	R:	AGAGAGATCAGTTGAGGTGGGA	
*gls2a*	F:	GCACGAATTAGGCAGTGAGC	XM_044211261.1
	R:	TGACGATAGCTCCGGCATTC	
*gls2b*	F:	ACGTATGCTGCGAACAGACT	XM_044219854.1
	R:	ACGGAGACCCTCTGCTACAT	
*rhbg*	F:	TGAGACGGATGCAAGCAAGT	XM_044207012.1
	R:	CTGGAAACTTGGGTAGCGGT	
*rhcg1*	F:	ACTTACCTTGCCCTCGCTTC	XM_044195378.1
	R:	AAACTCTGCTGCTGTTCCCA	
*rhcg2*	F:	GCTCTATCGGCCAAACCTGA	XM_044193958.1
	R:	CACAGGAAGAGGGTGCCAAT	
*rhag*	F:	TACCTGCGGTGTCCACAATC	XM_044170002.1
	R:	AGCAAACCCGAGGGATGAAG	
*slc9a3*	F:	GCAGTGCTCAGGCCAGATAAA	XM_044172873.1
	R:	AACCTCAGATTGTGTGCGAGT	
*nka*	F:	AAGTATTGGAGGGTCTGCCG	XM_044188659.1
	R:	AACTCCTGAATGGGTGCGTTA	
*β-actin*	F:	TGCGTGACATCAAGGAGAAGC	XM_044169301.1
	R:	GAGGAAGGAAGGCTGGAAGAG	

### 2.7 Data analysis

All data were analyzed using SPSS 22.0 software (SPSS Inc., USA). Prior to analysis, normality and homogeneity of variance were tested using the Shapiro-Wilk test and Levene’s test, respectively. A one-way analysis of variance (ANOVA) was performed to test for significance, followed by Duncan’s multiple comparisons test to compare timepoints and treatments (normoxia, hypoxia, hyperoxia). Pearson correlation analysis was performed to explore relationships between gene expression levels and corresponding physiological parameters. Data are presented as mean ± SD. A *P*-value of< 0.05 was considered to indicate a significant difference.

## 3 Results

### 3.1 Effects of DO levels on water quality indices and ammonia excretion rate

**Figure 1:**
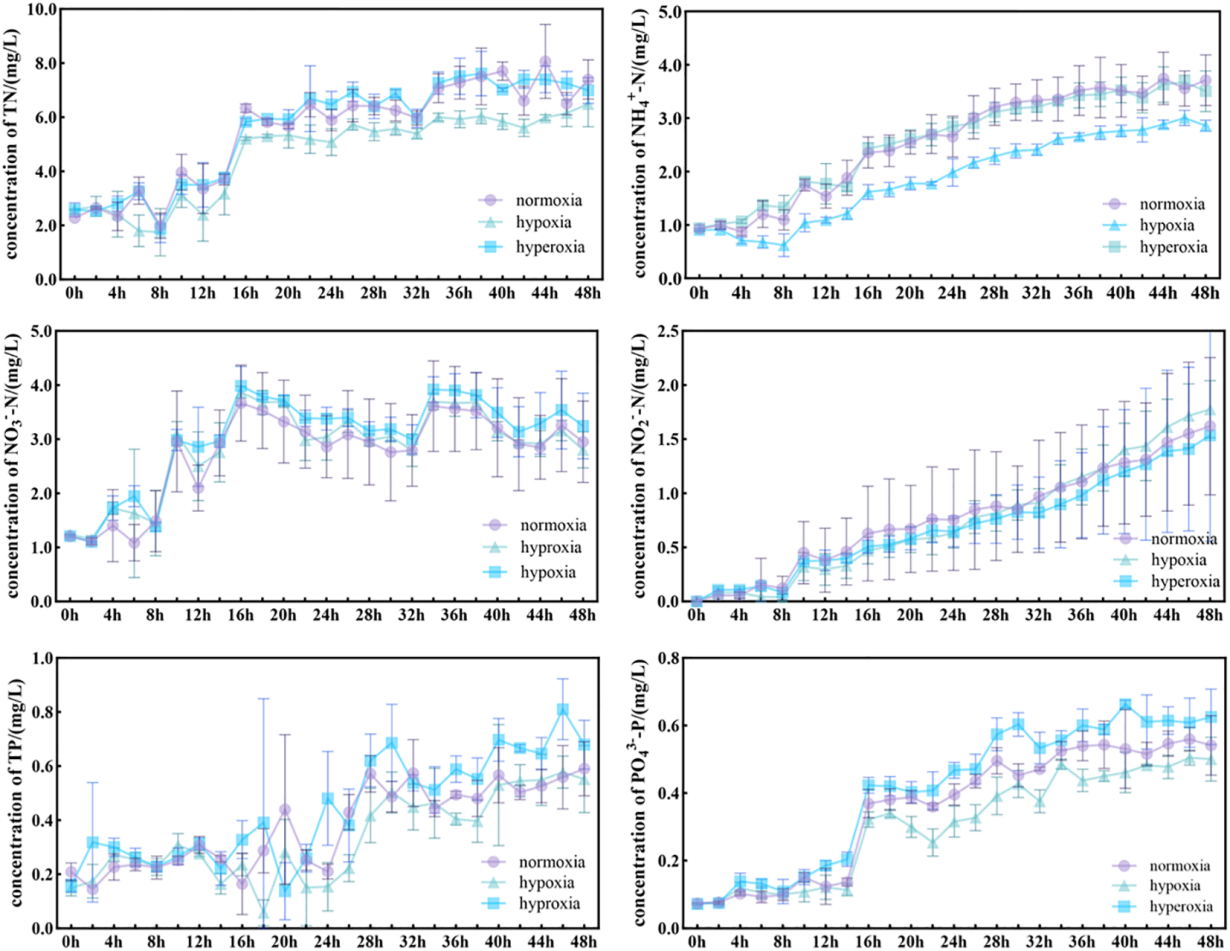
Dynamic changes of water quality parameters under different DO levels. Total nitrogen (TN); Nitrate nitrogen (NO_3_^-^-N); Ammonia nitrogen (NH_4_^+^-N); Nitrite nitrogen (NO_2_^-^-N); Phosphate (PO_4_³^-^-P); Total phosphorus (TP). Data are presented as mean ± SEM (n = 3).

No mortality was observed in any treatment group during the 48-h exposure period. During the 48-h exposure period, the concentrations of NH_4_^+^-N and NO_2_^-^-N in the water increased steadily from 0 h in all groups (**Figure 1**). The concentrations of TN and NO_3_^-^-N increased significantly between 8 and 16 hours, after which being stabilized. Meanwhile, The PO_4_^3-^-P content increased gradually from 12 h, while the TP content increased gradually from 24 h (**Figure 1**).

**Figure 2:**
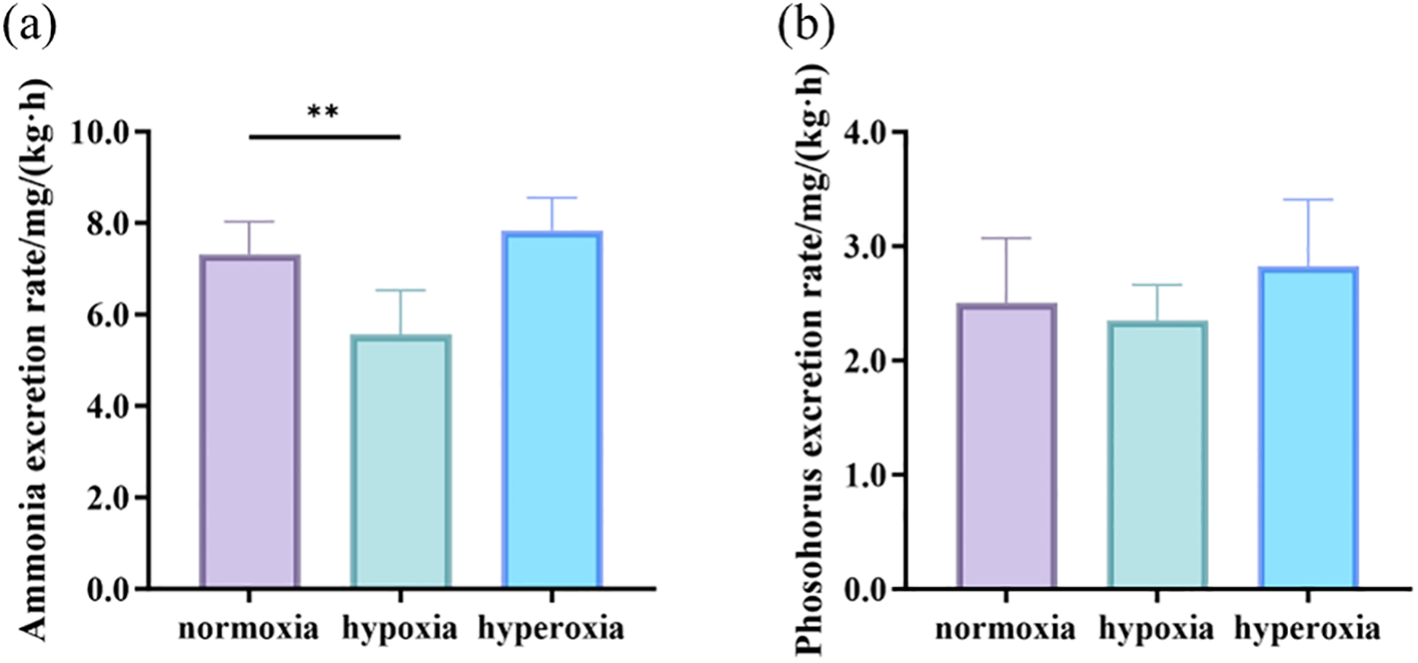
Effects of different DO levels on ammonia and phosphate excretion in mandarin fish. **(a)** Ammonia excretion rate; **(b)** Phosphorus excretion rate. Data are presented as mean ± SD (n = 6). Asterisks (*) indicate significant differences (*P*< 0.05).

For the effects of DO on water quality, the hypoxia group showed significantly lower water NH_4_^+^-N accumulation (reduced by 24.06% at 48 h) in the water, while PO_4_^3-^-P accumulation was lower after 16 h. There was no significant difference in ammonia nitrogen accumulation and ammonia excretion rate between the hyperoxia and normoxia groups, but the phosphate accumulation in the hyperoxia group was higher than that in the normoxia group from 12 h. No significant difference in phosphate excretion was observed among groups (**Figure 2**).

### 3.2 Effects of DO levels on the level of metabolites in plasma and liver

**Figure 3:**
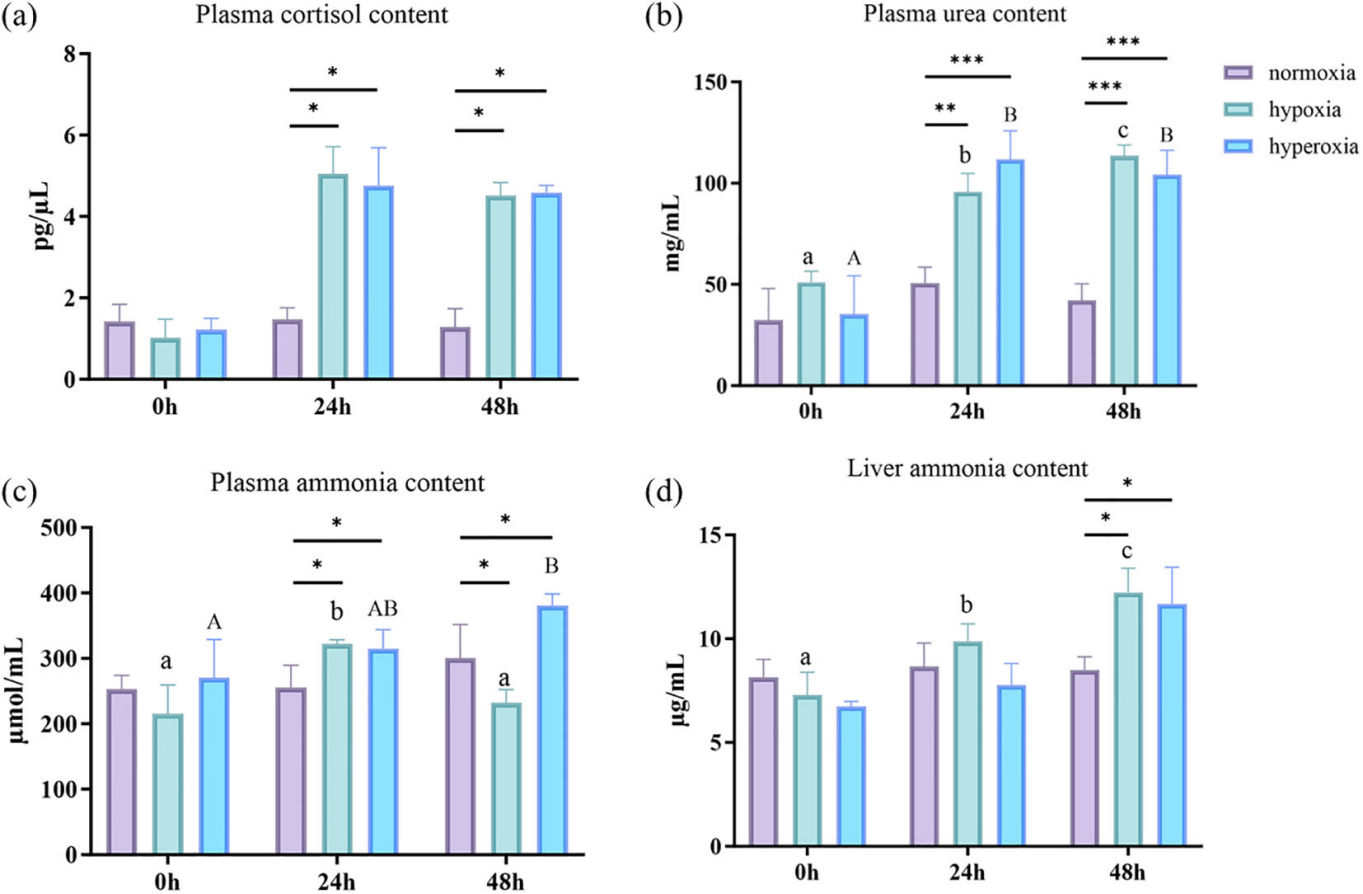
Effects of different DO levels on ammonia and urea contents in the plasma and liver. **(a)** plasma cortisol; **(b)** Plasma urea; **(c)** Plasma ammonia; **(d)**Liver ammonia. Data are presented as mean ± SD (n = 6). Asterisks (*) indicate significant differences among groups at the same time point (*P*< 0.05). Different letters indicate significant differences compared to 0 h within the same group (*P*< 0.05).

In the hypoxia group, the plasma cortisol levels were significantly elevated in both 24 h and 48 h (*P<* 0.05). The plasma ammonia levels were significantly higher at 24 h (peaked at 3.22 ± 0.37 μmol/mL) and significantly lower at 48 h (declined to 2.32 ± 0.47 μmol/mL) than in the normoxia group (**Figure 3**, *P*< 0.05). Exposure to hypoxia induced significantly higher plasma urea levels at both time points as compared to the normoxia group (**Figure 3**, *P*< 0.05). The liver ammonia content increased significantly with exposure time, being significantly higher than that in the normoxia group at 48 h (*P*< 0.05). As the exposure time increased, both plasma urea and liver ammonia levels accumulated significantly.

In the hyperoxia group, both the plasma ammonia and urea levels increased significantly as compared to the normoxia group at both timepoints (*P*< 0.05). The liver ammonia content was significantly higher than that in the normoxia group at 48 h (*P*< 0.05). For the time effects, the plasma ammonia content showed significantly higher level at 48 h, while the plasma urea content showed significantly higher levels at both exposure timepoints as compared to 0 h (*P<* 0.05). No significant differences in liver ammonia levels were observed between groups over time. Moreover, hyperoxia induced significant higher plasma cortisol levels in both timepoints (*P<* 0.05).

### 3.3 Effects of DO levels on antioxidant capacity in liver

**Figure 4:**
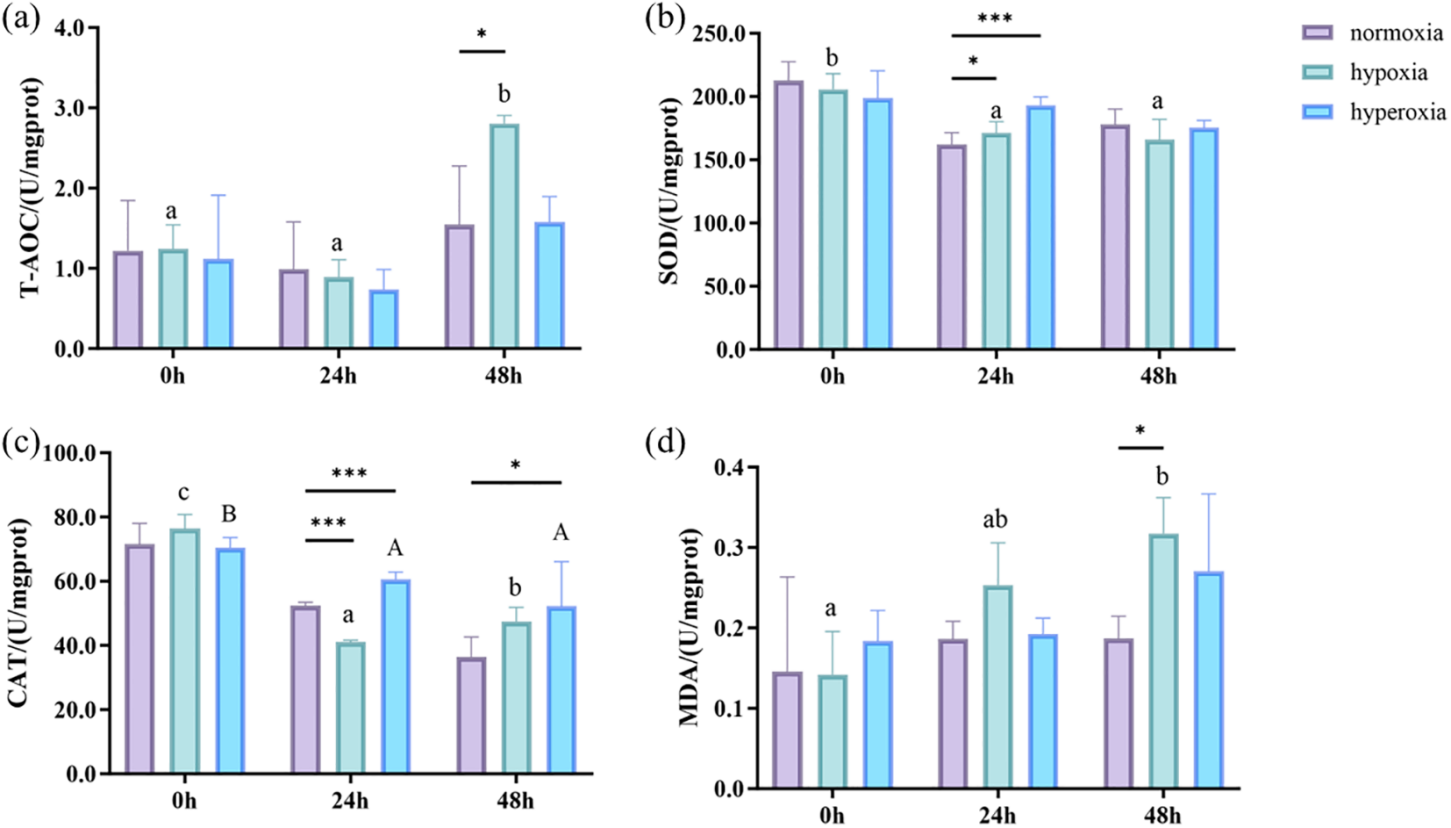
Effects of different DO levels on hepatic antioxidant parameters in mandarin fish. **(a)** Total antioxidant capacity (T-AOC); **(b)** Superoxide dismutase (SOD) activity; **(c)** Catalase (CAT) activity; **(d)** Malondialdehyde (MDA) content. Data are presented as mean ± SD (n = 6). Asterisks (*) indicate significant differences among groups at the same time point (*P*< 0.05). Different letters indicate significant differences compared to 0 h within the same group (*P*< 0.05).

Under hypoxic conditions, the activities of liver enzymes SOD and CAT were significantly lower than that in the normoxia group at 48 h and 24 h, respectively (**Figure 4**, *P*< 0.05). Meanwhile, the liver enzyme activities of T-AOC were significantly higher than that in the normoxia group at 48 h (*P*< 0.05). As the exposure time increased, the enzyme activities of SOD and CAT in the liver decreased significantly at both 24 h and 48 h (*P*< 0.05). In addition, the levels of MDA in the liver were highest at 48 h.

Under hyperoxic conditions, the activities of liver enzymes CAT (24 h) and SOD (both 24 h and 48 h) were significantly higher than in the normoxia group (*P*< 0.05). The activities of CAT decreased significantly at both 24 h and 48 h as the exposure expanded (*P*< 0.05). No significant differences were observed on the contents of MDA in the liver (*P* > 0.05) among groups.

### 3.4 Effects of DO levels on the expression of antioxidant-related genes in the liver

**Figure 5:**
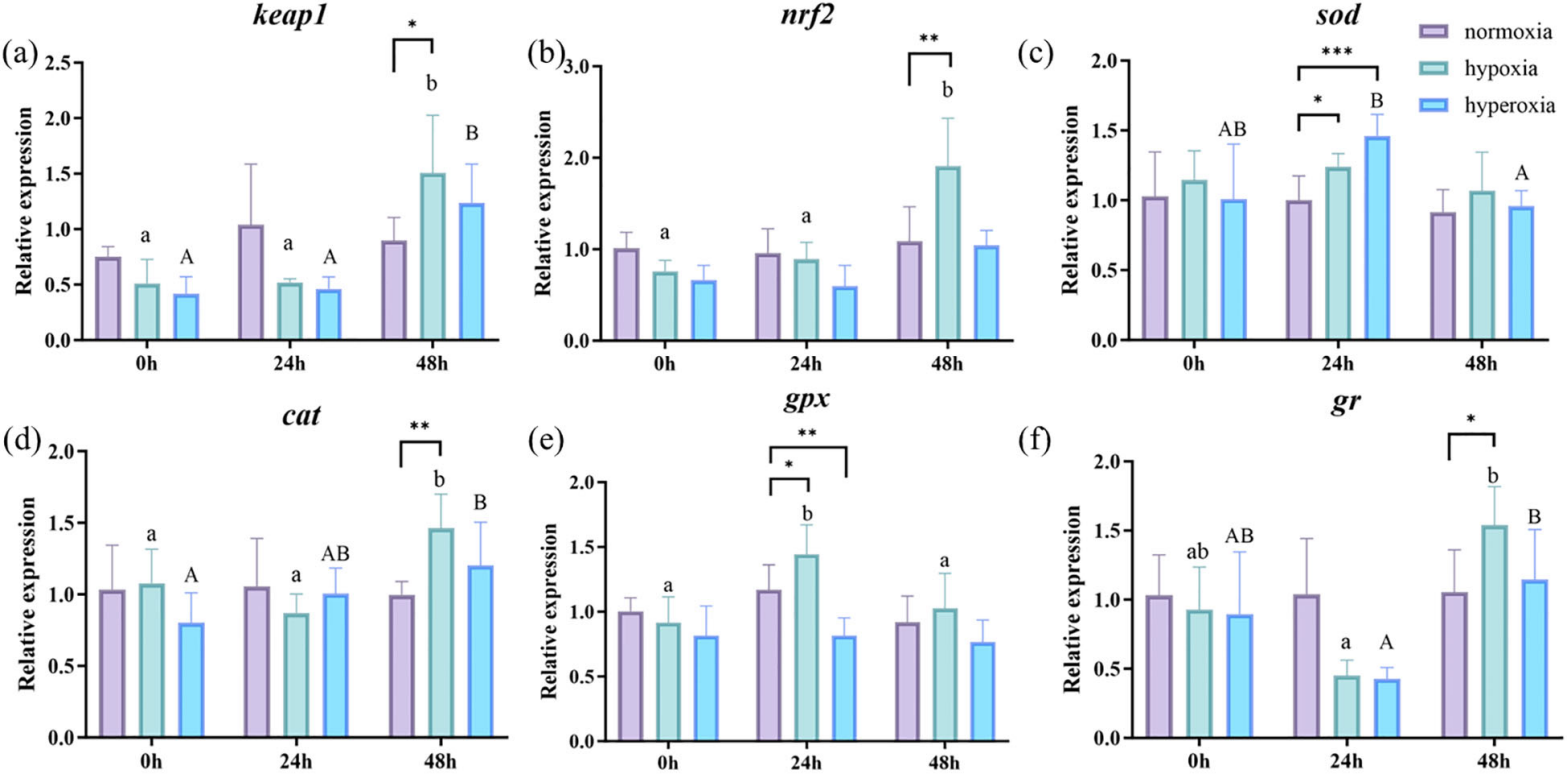
Effects of different DO levels on the expression of antioxidant-related genes in the liver of mandarin fish. Relative mRNA expression of **(a)** *keap1*; **(b)** *nrf2*; **(c)** *sod*; **(d)** *cat*; **(e)** *gpx*; **(f)** *gr*. Data are presented as mean ± SD (n = 6). Asterisks (*) indicate significant differences among groups at the same time point (*P*< 0.05). Different letters indicate significant differences compared to 0 h within the same group (*P*< 0.05).

In the hypoxia group, the transcriptional levels of *nrf2* and *keap1* in the liver were significantly higher than in the normoxia group at 48 h (**Figure 5**, *P*< 0.05). The hepatic gene expression of *sod* and *gpx* was significantly higher than that in the normoxia group at 24 h (*P*< 0.05). The expression of *cat* and *gr* first decreased and then increased, reaching levels that were significantly higher than those in the normoxia group at 48 h (*P*< 0.05).

In the hyperoxia group, the mRNA levels of *keap1* were highest at 48 h compared to the normoxia group (*P*< 0.05). The expression of *gr* and *sod* showed opposite trends, but no significant differences were found at 24 h or 48 h compared to 0 h. In addition, the expression of *cat* increased with the exposure duration, reaching the highest level at 48 h. There were no significant differences in *nrf2* and *gpx* expression among groups (*P* > 0.05).

### 3.5 Effects of DO levels on histological observation of liver and gill

**Figure 6:**
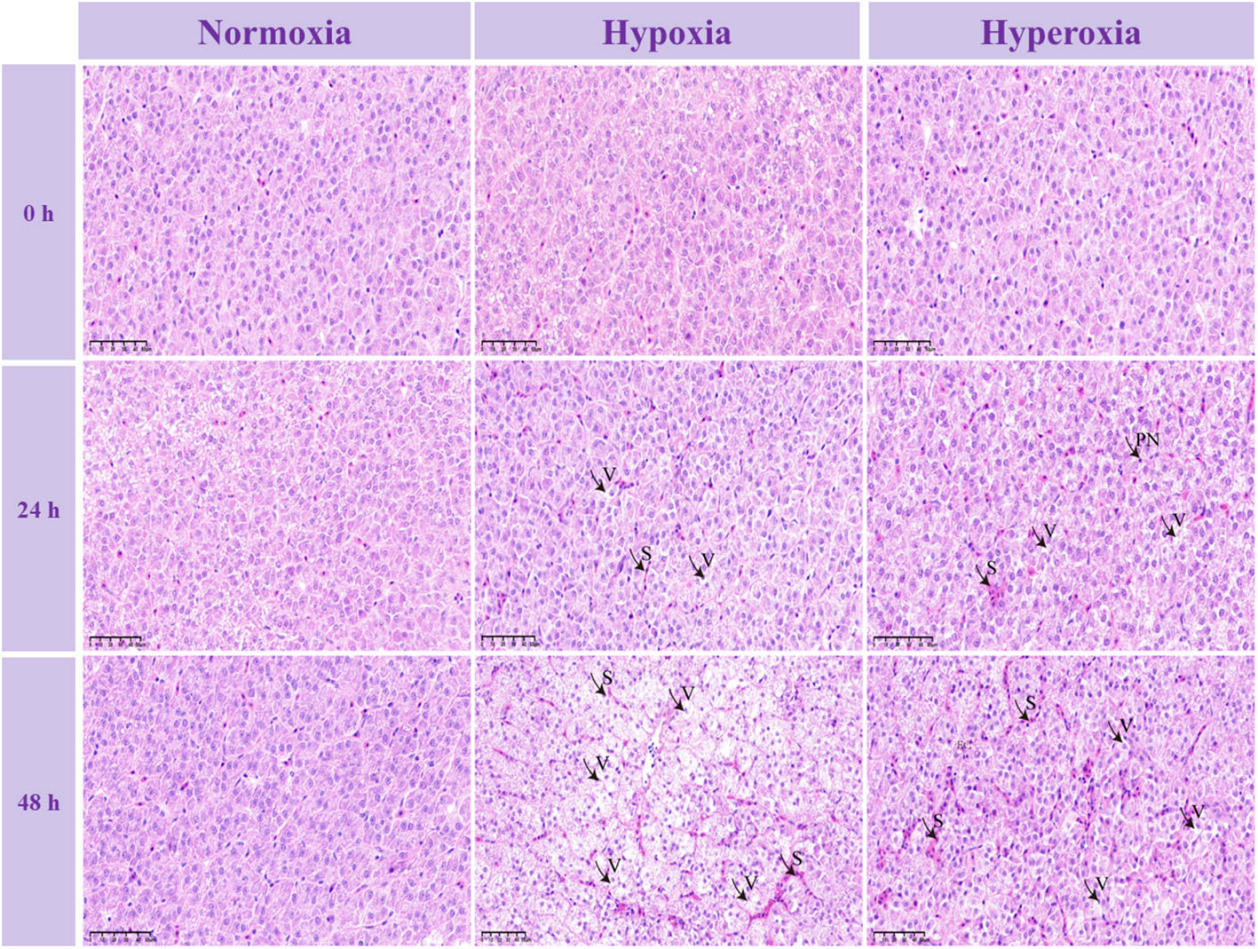
Histological observation of liver in mandarin fish exposed to different DO levels for 48 h. S, sinusoidal; V, vacuolization. Scale bar = 50 μm.

Histological examination of the liver in the normoxia group displayed a well-organized structure, with regular-shaped hepatocytes and tightly packed, spherical nuclei centered within the cytoplasm (**Figure 6**). Exposure to both hypoxia and hyperoxia induced nuclear displacement and cellular vacuolization.

**Figure 7:**
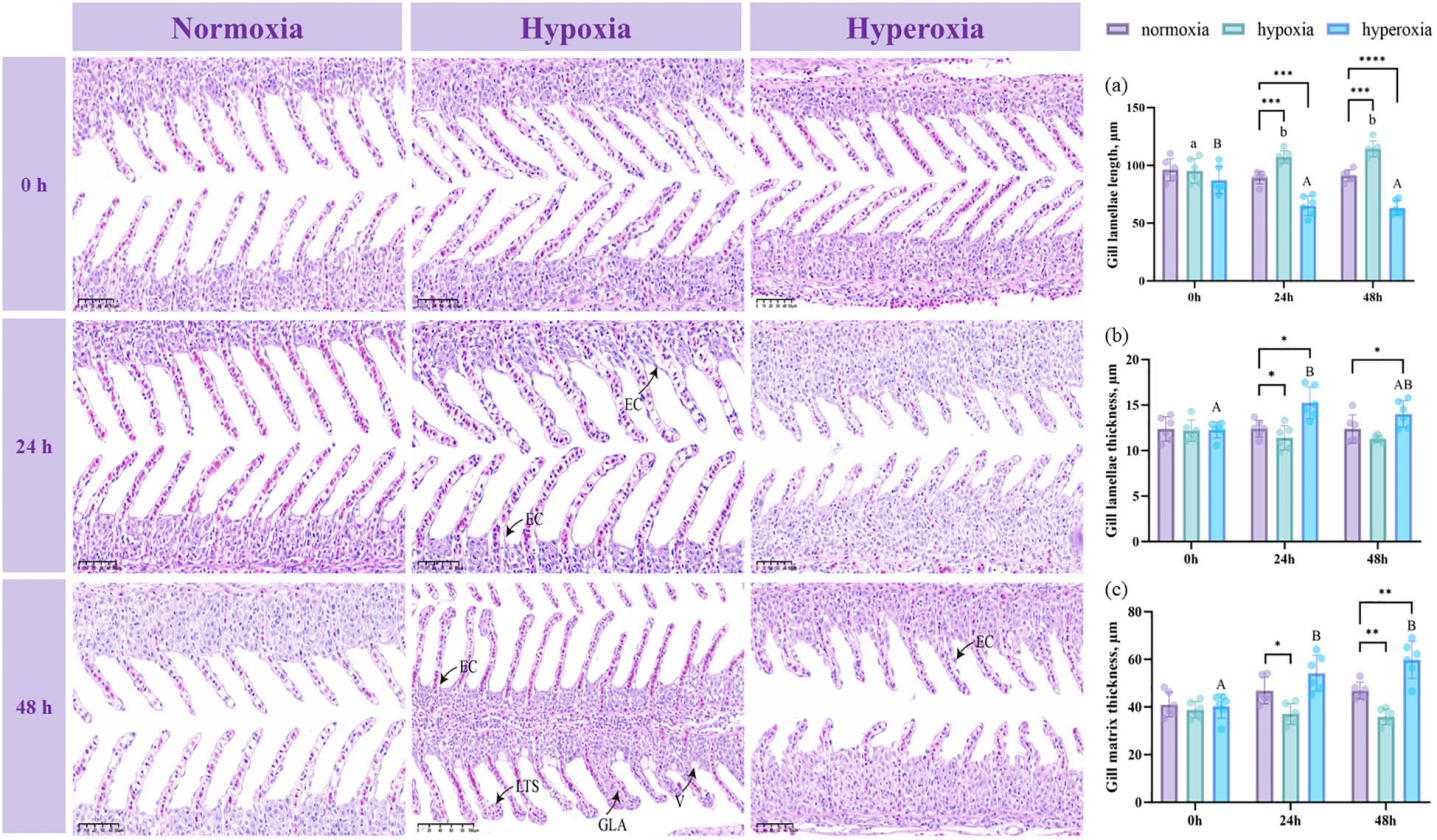
Histological observation of gill in mandarin fish exposed to different DO levels for 48 h. **(a)** Gill lamellae length; **(b)** Gill lamellae thickness; **(c)** Gill matrix thickness. EC, epithelial cell; GLA, gill lamellar adhesion; LTS, lamellar tip swelling; V, vacuoles. Scale bar = 50 μm.

The gill tissues in the normoxia and hyperoxia groups were structurally intact, with a large number of neatly and closely arranged gill lamellae visible on the gill filaments (**Figure 7**). In the hypoxia group, however, the branchial filament structure was abnormal, with atrophied and shorter filaments on both sides. No significant differences in branchial structure were observed in the hyperoxia group with the time of exposure.

### 3.6 Effects of DO levels on the expression of ammonia production and conversion -related genes

**Figure 8:**
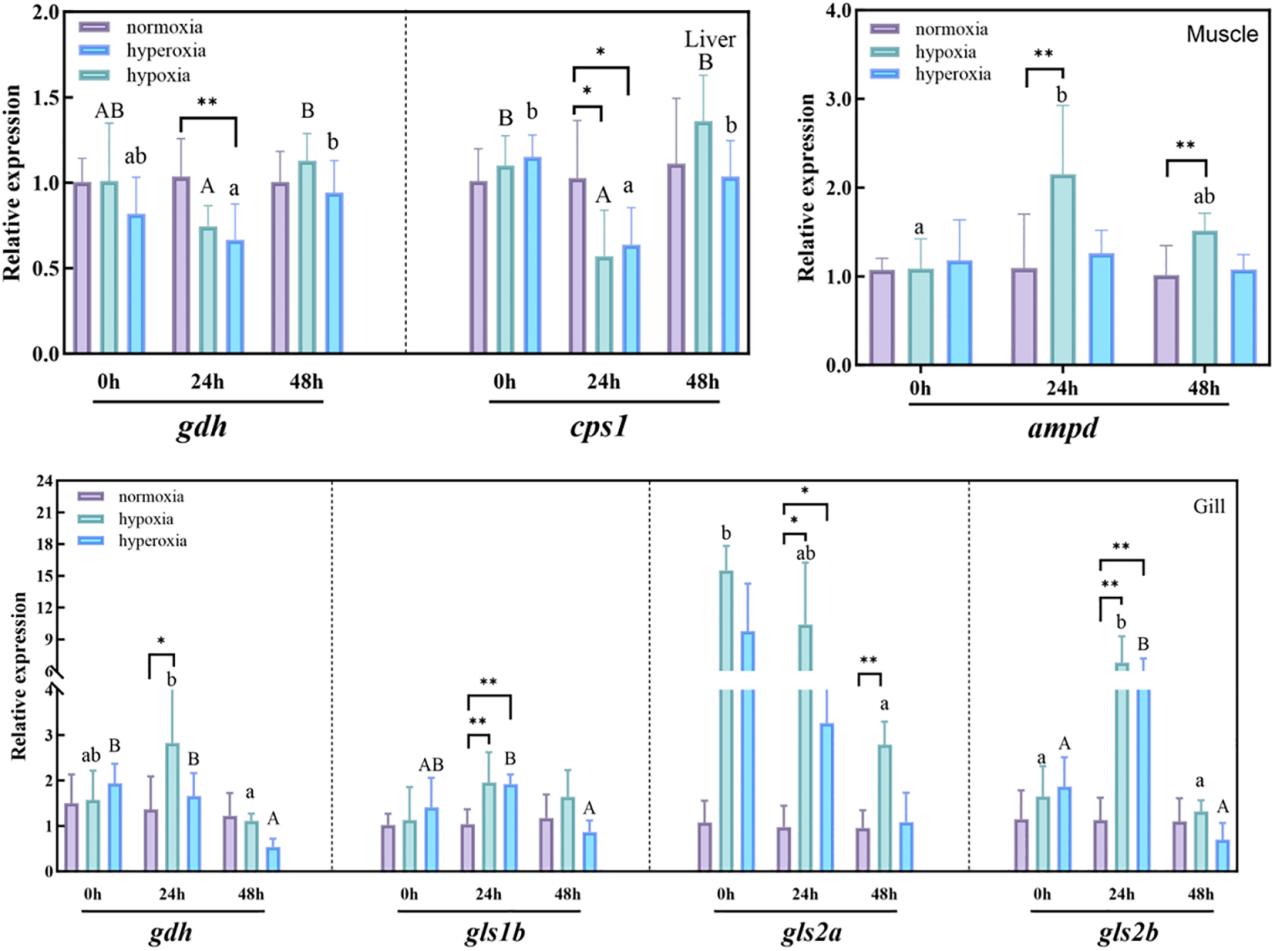
Effects of DO levels on the expression of ammonia production and conversion-related genes in mandarin fish. Data are presented as mean ± SD (n = 6). Asterisks (*) indicate significant differences among groups at the same time point (*P*< 0.05). Different letters indicate significant differences compared to 0 h within the same group (*P*< 0.05).

In the liver, the relative expression of liver *gdh* and *cps1* first decreased and then increased in both the hypoxia and hyperoxia groups, being significantly lower by 26.18% than those in the normoxia group at 24 h (**Figure 8A**, *P*< 0.05).

In the muscle, the expression of *ampd* first increased and then decreased in the hypoxia group, being significantly higher than that in the normoxia group at 24 h and 48 h (**Figure 8B**, *P*< 0.05). Hyperoxia had no significant effect on *ampd* expression (P > 0.05) (**Figure 8B**, *P* > 0.05).

In the gill, the expression of *gdh* first increased and then decreased in the hypoxia group, being significantly higher than that in the normoxia group at 24 h (**Figure 8C**, *P*< 0.05). In the hyperoxia group, the expression of *gdh* was significantly decreased at 48 h (*P*< 0.05). The expressions of *gls1b* and *gls2b* in both hypoxia and hyperoxia groups first increased and then decreased, being significantly higher than those in the normoxia group at 24 h (*P*< 0.05). The expression of *gls2a* decreased significantly with the hypoxia stress duration (*P*< 0.05).

### 3.7 Effects of DO levels on the expression of ammonia excretion-related genes in the gills

**Figure 9:**
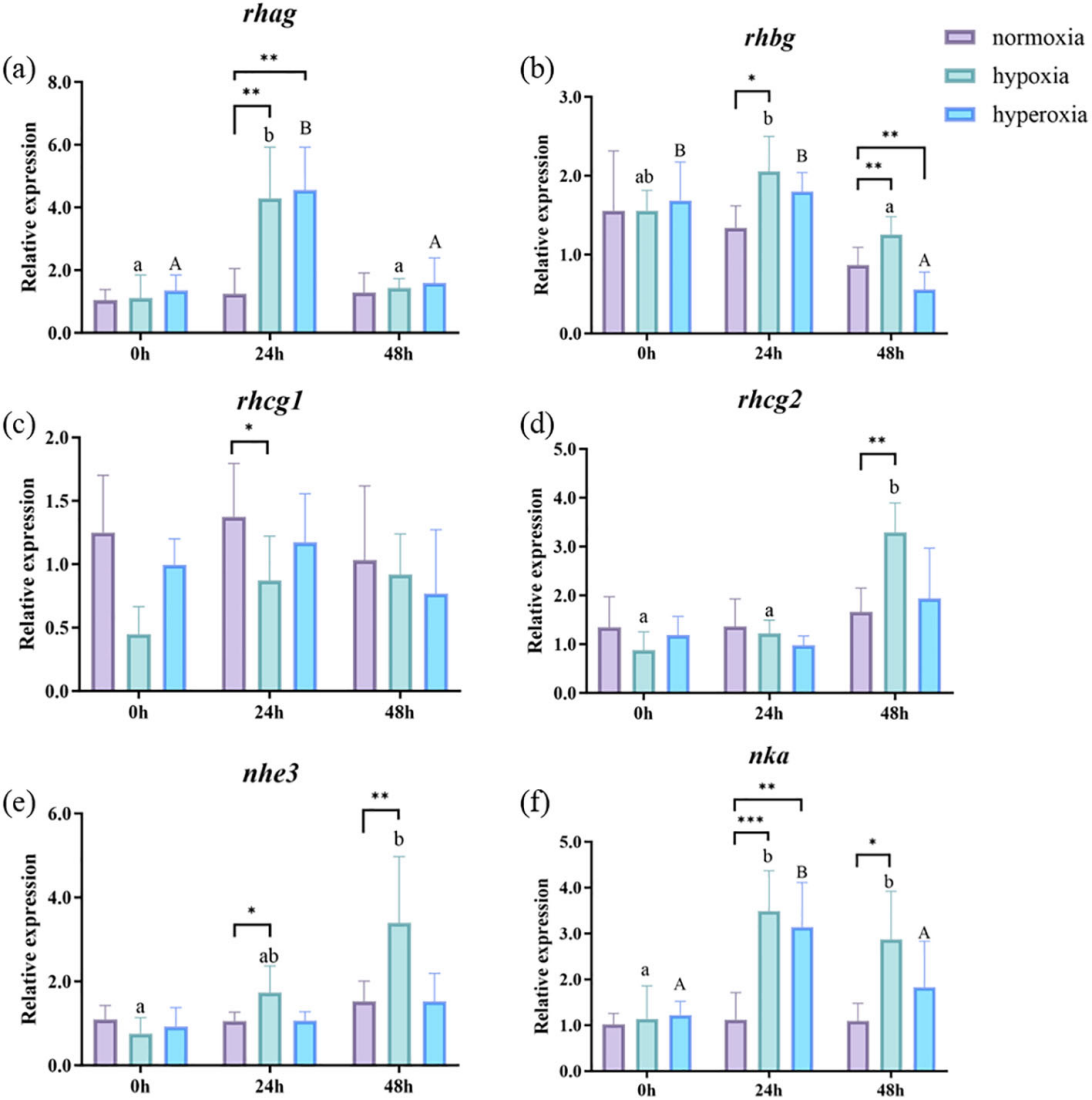
Effects of DO levels on the expression of ammonia excretion-related genes in the gills. Relative mRNA expression of **(a)** *rhag*; **(b)** *rhbg*; **(c)** *rhcg1*; **(d)** *rhcg2*; **(e)** *nhe3*; **(f)** *nka*. Data are presented as mean ± SD (n = 6). Asterisks (*) indicate significant differences among groups at the same time point (*P*< 0.05). Different letters indicate significant differences compared to 0 h within the same group (*P*< 0.05).

In the hypoxia group, the expression of *rhag* and *rhcg1* was significantly higher than that in the normoxia group at 24 h (**Figure 9**, *P*< 0.05). The expression of *rhbg*, *nhe3*, and *nka* were significantly higher at both 24 h and 48 h compared to the normoxia group (*P*< 0.05). Regarding time effects, *rhcg2* and *nhe3* expression levels were significantly higher at 48 h than at 0 h (*P*< 0.05).

In the hyperoxia group, the expression of *rhag* and *nka* were significantly higher, while the *rhbg* expression were significantly lower than those in the normoxia group at 24 h (*P*< 0.05). No significant differences were found in the expression of *rhcg1*, *rhcg2*, and *nhe3* among groups (*P* > 0.05). To visualize the overall expression patterns of all target genes in mandarin fish under different DO levels, a heatmap was generated (**Supplementary Figure S1**).

### 3.8 Correlation analysis between gene expression and physiological parameters

Pearson correlation analysis revealed a significant positive correlation between cps1 expression and plasma urea across all samples (r = 0.857, *P*< 0.001, n = 54), particularly in hyperoxia (r = 0.896, *P*< 0.001, n = 18) and hypoxia (r = 0.883, P< 0.001, n = 18), but not in normoxia (r = -0.196, *P* = 0.196, n = 18) (**Supplementary Table 1**). Conversely, *gdh* expression was negatively correlated with liver ammonia overall (r = -0.780, *P*< 0.001, n = 54), most strongly in hyperoxia (r = -0.932, *P*< 0.001, n = 18), followed by hypoxia (r = -0.562, *P* = 0.015, n = 18), with no correlation in normoxia (r = 0.075, *P* = 0.768, n = 18). No significant correlation was found between rhcg2 expression and ammonia excretion rate at 48 h, either overall (r = -0.156, *P* = 0.537, n = 18) or within any treatment group (*P* > 0.05 for all).

## 4 Discussion

### 4.1 DO-mediated antioxidant defense as an adaptive strategy in tissue damage and ammonia excretion

Fluctuations in dissolved oxygen and accumulation of ambient ammonia are common and interrelated stressors in intensive aquaculture, both capable of inducing oxidative stress in fish (Wu, 2002; Zhao et al., 2020; Lushchak, 2011). Plasma cortisol levels, a key indicator of physiological stress, were significantly elevated in both hypoxia and hyperoxia groups at both 24 h and 48 h (**Figure 3d**, P< 0.05), confirming that both DO conditions induced a stress response in *S. chuatsi*. The Nrf2/Keap1 pathway is a key regulatory axis of the antioxidant defense system, modulating the expression of enzymes that scavenge reactive oxygen species (Kobayashi and Yamamoto, 2006; Li et al., 2022; Bian et al., 2025). In this study, distinct oxidative states and adaptive antioxidant responses were observed in *S. chuatsi* under different DO regimes. Exposure to hypoxia induced oxidative stress in *S. chuatsi*, as evidenced by the progressively elevated MDA content, suppressed SOD/CAT activities, and histopathological liver damage. In response, the Nrf2/Keap1 signaling pathway was activated, with upregulation of *nrf2, keap1* and the downstream genes (*gr*, *cat*) at 48 h compared to the normoxia group. These results suggested that *S. chuatsi* enhanced its antioxidant capacity via the Nrf2/Keap1 pathway to mitigate oxidative damage under hypoxia, which is consistent with previous study on yellow catfish (*Pelteobagrus fulvidraco*) under hypoxic conditions (Wang et al., 2021). In contrast, hyperoxia did not elicit significant lipid peroxidation as indicated by the stable MDA levels in the liver. However, the hepatic activities of SOD and CAT were significantly elevated, and the expression of *sod* was upregulated at 24 h as compared to the hypoxia group, indicating enhanced antioxidant capacity in *S. chuatsi* under hyperoxia.

Oxidative stress in fish typically results in retarded growth performance, metabolic disorders and tissue damage. In teleosts, the gills are the primary site for both gas exchange and ammonia excretion, accounting for over 80% of total nitrogenous waste removal (Bucking, 2017). Hypoxia-induced oxidative stress has been linked to structural abnormalities in the gills, including filament atrophy. Such damage is known to impair branchial integrity and function, thereby compromising the efficiency of ammonia excretion (Zimmer, 2024). Consistent with this, the observed gill alterations under hypoxia likely contributed to a reduction in branchial excretory capacity. This is reflected in the significantly lower ammonia accumulation in the water, a decreased ammonia excretion rate, and the concurrently rising hepatic ammonia levels in *S. chuatsi* in the hypoxia group. Therefore, the tailored antioxidant responses in *S. chuatsi* under both hypoxia and hyperoxia can be interpreted as a crucial first-line defense. By alleviating oxidative stress and potentially limiting gill damage, this response helps to preserve the function of the excretory organ. This foundational adaptation works in concert with subsequent metabolic reprogramming (e.g., altered ammonia production and conversion) to maintain overall ammonia homeostasis. This finding highlights the close link between antioxidant defense and ammonia pollution adaptation, providing a physiological rationale for leveraging DO management to mitigate ammonia pollution.

### 4.2 Hypoxia-driven ammonia metabolic regulation for pollution mitigation

Reducing ammonia production is an important strategy for fish to cope with ammonia accumulation and can also reduce the environmental impact of aquaculture (Ip et al., 2001). Glutamate dehydrogenase (GDH) is a key enzyme involved in hepatic ammonia production via amino acid catabolism (Gaspar et al., 2018). In the present study, the plasma ammonia (PA) content decreased significantly at 48 h after peaking at 24 h, indicating that *S. chuatsi* can initiate adaptive strategies to cope with high ammonia pressure induced by hypoxia exposure. The hepatic *gdh* expression was significantly downregulated at 24 h under hypoxic, indicating that *S. chuatsi* reduces liver-derived ammonia production by suppressing amino acid degradation to alleviate high ammonia pressure. Consistently, *gdh* expression showed a significant negative correlation with liver ammonia content under hypoxia and hyperoxia. Adenosine deaminase (AMPD) plays a vital role in the indirect deamination, providing an energy supply for the muscles (Yang et al., 2024). The upregulation of muscle *ampd* at 24 h under hypoxic conditions suggested enhanced deamination to meet the increased energy demands of environmental adaptation. The concurrent reduction in ammonia production in the liver and upregulation in muscle ensures an adequate energy supply while ultimately suppressing ammonia release, which is an important aspect of metabolic plasticity.

Converting ammonia into less toxic metabolites can reduce its toxicity in fish and the amount excreted into the environment. When ammonia accumulates to excessive levels, some of it is diverted to the liver for conversion into non-toxic nitrogenous compounds, such as urea or glutamine (Campbell, 1997). Glutamine synthesis is considered the primary ammonia-detoxification pathway in most teleosts (Chew and Ip, 2014). For instance, the largemouth bass (*Micropterus salmoides*) primarily convert ammonia to glutamine in response to ammonia stress (Zou et al., 2023). In contrast, our study found that plasma urea (PU) content increased significantly in the hypoxia group, while the expression of glutaminase (*gls2a*) in the gills decreased significantly at 48 h compared to the 0 h. Carbamyl phosphate synthase 1 (CPS1) is a rate-limiting enzyme in the ornithine-urea cycle, which converts ammonia to urea (Zhang et al., 2022). In yellow catfish (*Pelteobagrus fulvidraco*), acute ammonia stress similarly upregulates hepatic cps1 expression and activates the urea cycle (Zhang et al., 2022). In the present study, the expression of *cps1* in the liver was initially suppressed at 24 h but subsequently increased by 48 h, coinciding with a significant decline in plasma ammonia. Therefore, the findings suggested that under hypoxic stress and concomitant internal ammonia accumulation, *S. chuatsi* preferentially shunts excess ammonia toward urea synthesis rather than glutamine formation. This was further supported by the significant positive correlation between cps1 expression and plasma urea under hypoxia and hyperoxia.

The ultimate way to eliminate internal ammonia is to enhance ammonia excretion capacity. In freshwater fish, RH glycoproteins (Rhag, Rhbg, Rhcg1, Rhcg2) and Na^+^/H^+^ exchanger 3 (NHE3) play key roles in branchial ammonia excretion (Ip and Chew, 2010a). Generally, Rhag generally facilitates the uptake of ammonia from the blood into the basolateral side of gill cells and Rhbg mediates its intracellular translocation of ammonia towards the apical membrane. At the apical surface, ammonia is actively excreted into the water via the combined action of the NHE3 and the apical ammonia transporter Rhcg2 (Ip and Chew, 2010a; Parvathy et al., 2023). In this study, hypoxia upregulated *rhag* and *rhbg* at 24 h, and *rhcg2* and *nhe3* at 48 h. However, this molecular upregulation should be viewed as a compensatory response to the concurrent gill structural damage, which impairs excretory surface area. Consequently, despite increased transporter expression, the net ammonia excretion rate in the hypoxia group was significantly lower than in the normoxia group, and water NH_4_^+^-N accumulation was reduced by 24.06%. Thus, the reduced net ammonia release under hypoxia results from an integrated strategy: (i) reduced hepatic ammonia production (downregulated *gdh*), (ii) enhanced conversion to urea (upregulated cps1 and elevated plasma urea), (iii) lower plasma ammonia at 48 h decreasing passive NH_3_ diffusion, and (iv) compensatory but insufficient upregulation of branchial transporters.

### 4.3 Hyperoxia enhances ammonia tolerance of *S. chuatsi* through metabolic conversion

Supplemental oxygen is commonly employed in modern intensive aquaculture to prevent oxygen deficiency. Supersaturated oxygen has previously been found to have no beneficial effect on fish growth, but it can elevate the tolerance to ammonia toxicity (Dong et al., 2013). In the present study, exposure to hyperoxia had no significant impact on the accumulation of ammonia in the water or the excretion rate of ammonia, suggesting that the ability to excrete ammonia was not impaired. However, hyperoxia exposure induced significant increases in plasma and ammonia levels at 48 h, which was consistent with reports on mandarin fish subjected to prolonged oxygen enrichment (Yang et al., 2024). Concerning the endogenous ammonia production pathways, no significant differences were found on the transcriptional levels of *ampd* in the muscle as compared to the normoxia group. However, the relative expressions of ammonia generation-related gene *gdh* and *gls2a* in the liver were significantly downregulated at 24 h compared to the normoxia group, implying reduced ammonia generation under hyperoxia. Moreover, the PU content was significantly higher in the hyperoxia group at both 24 h and 48 h, suggesting that *S. chuatsi* converts ammonia into urea to overcome high ammonia toxicity.

It is generally recognized that certain fish, such as the yellow catfish (*Pelteobagrus fulvidraco*), exhibit enhanced tolerance to ammonia under supersaturated oxygen conditions (Yang et al., 2024). Regarding ammonia excretion under hyperoxia, the expression of *rhag* and *nka* in the gills of *S. chuatsi* was significant upregulated 24 h, facilitating the transport of ammonia from the blood to the epithelial cells. Nevertheless, the expression levels of *rhbg*, *rhcg1*, *rhcg2*, and *nhe3* remained unchanged across the different groups. These results indicated that under hyperoxic conditions, *S. chuatsi* counteracted elevated ammonia stress by reducing ammonia production, enhancing ammonia conversion to urea, and increasing ammonia transport to the gills, without significantly increasing the final excretory flux. However, this strategy does not significantly influence hepatic ammonia accumulation or blood ammonia levels. In addition, the activities of antioxidant enzymes such as SOD and CAT were stimulated in the liver of *S. chuatsi* under oxygen-enriched conditions, so were the undamaged liver histology results, indicating the homeostasis of liver health under hyperoxia. Taken together, the reduction in ammonia production and the increase in urea conversion did not reduce the ammonia level in the liver, but maintained its health status, suggesting hyperoxia enhances ammonia tolerance despite elevated internal ammonia, as the fish can withstand higher ammonia loads without tissue damage.

Limitations and future perspectives. This study did not evaluate ammonia toxicity endpoints (behavior and growth), and the 48-h exposure experiment only showed acute responses. Gut microbiota was not assessed, and functional validations (enzyme activity, protein abundance, transporter localization, metabolic flux) are lacking. Additionally, our findings are based on gene expression data; further investigation via enzyme inhibition or gene knockout approaches should be investigated in the future. Future studies incorporating these aspects are needed to validate and extend the findings.

## 5 Conclusions

**Figure 10:**
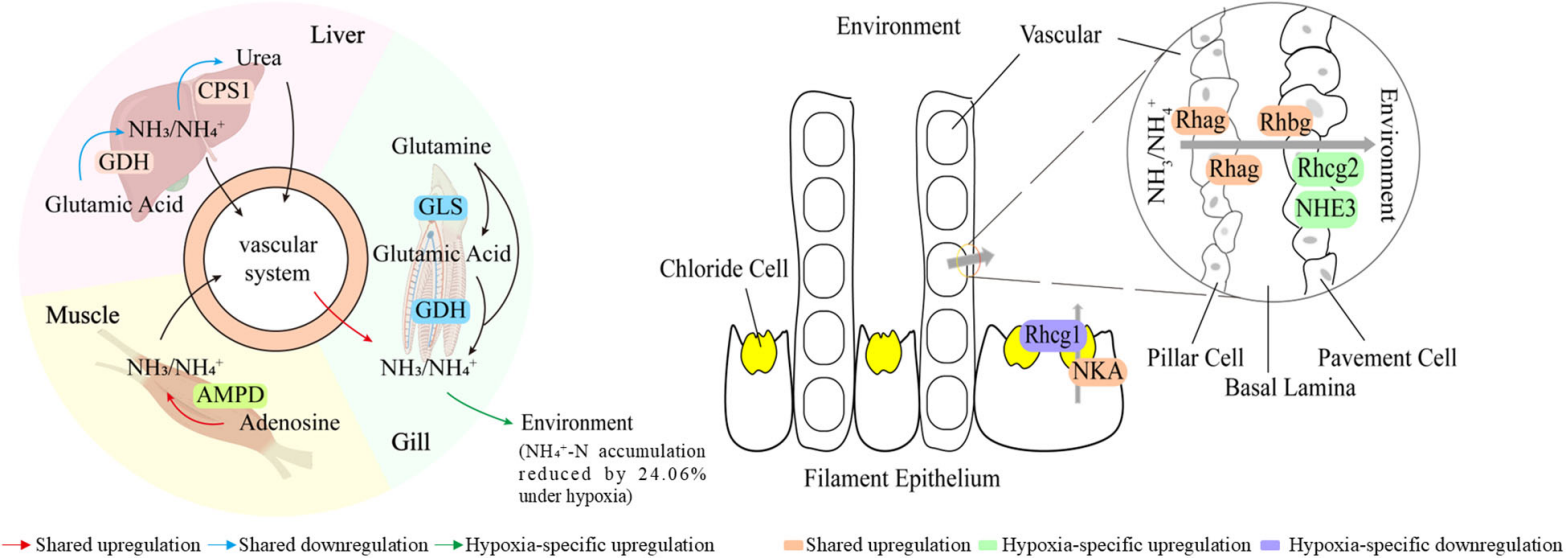
Proposed mechanistic model of DO-mediated ammonia metabolism plasticity in mandarin fish. Changes are shown relative to normoxia. Red: upregulated under both hypoxia and hyperoxia; blue: downregulated under both hypoxia and hyperoxia; green: downregulated under hypoxia only.

This study elucidated that dissolved oxygen critically regulated antioxidant defense and ammonia metabolism in mandarin fish, revealing DO-mediated metabolic plasticity as a biological strategy for mitigating aquaculture wastes. Hypoxia triggered oxidative stress and structural alterations to the gills, resulting in a lower rate of ammonia excretion. Meanwhile, hypoxia downregulated ammonia production, enhanced ureogenesis, and attempted to maintain excretion via compensatory upregulation, ultimately decreasing water NH_4_^+^-N by 24.06%. In contrast, hyperoxia promoted ammonia tolerance by increasing urea conversion and antioxidant capacity, while maintaining tissue integrity, without enhancing excretion (see proposed mechanistic model in **Figure 10**). These findings demonstrated that targeted DO modulation can effectively leverage fish’s intrinsic physiology to mitigate nitrogen pollution.

## Data Availability

The raw data supporting the conclusions of this article will be made available by the authors, without undue reservation.
