# Supplementary material for: Dissolved oxygen levels differentially regulate ammonia metabolism plasticity in mandarin fish (Siniperca chuatsi)
Source: Front Physiol. 2026 Aug 4;17:1912438. doi: 10.3389/fphys.2026.1912438 (PMC13481236; doi:10.3389/fphys.2026.1912438)
Supplement: Supplementary file 1 [file DataSheet1.docx]

**Supplementary Materials**

**Supplementary Fig. S1. Heatmap of global gene expression patterns for 15 target genes in mandarin fish under different DO levels (normoxia, hypoxia, hyperoxia) and time points (0, 24, 48 h).** Expression values are log₂-transformed fold changes relative to 0 h. Red indicates upregulation; blue indicates downregulation. Hierarchical clustering was performed using Pearson correlation and average linkage.

**Supplementary Table S1. Pearson correlation coefficients between gene expression and physiological parameters**

| Variable pair | Group | r | *P*-value | n | Significance |
| --- | --- | --- | --- | --- | --- |
| ***rhcg2* expression vs. ammonia excretion rate** | **All** | **-0.156** | **0.537** | **18** | **NS** |
| *rhcg2* expression vs. ammonia excretion rate | Normoxia | -0.169 | 0.750 | 6 | NS |
| *rhcg2* expression vs. ammonia excretion rate | Hyperoxia | -0.226 | 0.667 | 6 | NS |
| *rhcg2* expression vs. ammonia excretion rate | Hypoxia | 0.190 | 0.719 | 6 | NS |
| ***cps1* expression vs. plasma urea** | **All** | **0.857** | **<0.001** | **54** | ******* |
| *cps1* expression vs. plasma urea | Normoxia | -0.196 | 0.196 | 18 | NS |
| *cps1* expression vs. plasma urea | Hyperoxia | 0.896 | <0.001 | 18 | *** |
| *cps1* expression vs. plasma urea | Hypoxia | 0.883 | <0.001 | 18 | *** |
| ***gdh* expression vs. liver ammonia** | **All** | **-0.780** | **<0.001** | **54** | ******* |
| *gdh* expression vs. liver ammonia | Normoxia | 0.075 | 0.768 | 18 | NS |
| *gdh* expression vs. liver ammonia | Hyperoxia | -0.932 | <0.001 | 18 | *** |
| *gdh* expression vs. liver ammonia | Hypoxia | -0.562 | 0.015 | 18 | * |
